# Novel spatiotemporal processing tools for body-surface potential map signals for the prediction of catheter ablation outcome in persistent atrial fibrillation

**DOI:** 10.3389/fphys.2022.1001060

**Published:** 2022-09-29

**Authors:** Anna McCann, Adrian Luca, Patrizio Pascale, Etienne Pruvot, Jean-Marc Vesin

**Affiliations:** ^1^ Applied Signal Processing Group, Department of Electrical Engineering, Swiss Federal Institute of Technology, Lausanne, Switzerland; ^2^ Service of Cardiology, Lausanne University Hospital, Lausanne, Switzerland

**Keywords:** atrial fibrillation, catheter ablation, body surface potential mapping, spatiotemporal analysis, outcome stratification

## Abstract

**Background:** Signal processing tools are required to efficiently analyze data collected in body-surface-potential map (BSPM) recordings. A limited number of such tools exist for studying persistent atrial fibrillation (persAF). We propose two novel, spatiotemporal indices for processing BSPM data and test their clinical applicability through a comparison with the recently proposed non-dipolar component index (NDI) for prediction of single-procedure catheter ablation (CA) success rate in persAF patients.

**Methods:** BSPM recordings were obtained with a 252-lead vest in 13 persAF patients (8 men, 63 ± 8 years, 11 ± 13 months sustained AF duration) before undergoing CA. Each recording was divided into seven 1-min segments of high signal quality. Spatiotemporal ventricular activity (VA) cancellation was applied to each segment to isolate atrial activity (AA). The two novel indices, called error-ratio, normalized root-mean-square error (ER_NRMSE_) and error-ratio, mean-absolute error (ER_ABSE_), were calculated. These indices quantify the capacity of a subset of BSPM vest electrodes to accurately represent the AA, and AA dominant frequency (DF), respectively, on all BSPM electrodes over time, compared to the optimal principal component analysis (PCA) representation. The NDI, quantifying the fraction of energy retained after removal of the three largest PCs, was also calculated. The two novel indices and the NDI were statistically compared between patient groups based on single-procedure clinical CA outcome. Finally, their predictive power for univariate CA outcome classification was assessed using receiver operating characteristic (ROC) analysis with cross-validation for a logistic regression classifier.

**Results:** Patient clinical outcomes were recorded 6 months following procedures, and those who had an arrhythmia recurrence at least 2 months post-CA were defined as having a negative outcome. Clinical outcome information was available for 11 patients, 6 with arrhythmia recurrence. Therefore, a total of 77 1-min AA-BSPM segments were available for analysis. Significant differences were found in the values of the novel indices and NDI between patients with arrhythmia recurrence post-ablation and those without. ROC analysis showed the best CA outcome predictive performance for ER_NRMSE_ (AUC = 0.77 ± 0.08, sensitivity = 76.2%, specificity = 84.8%).

**Conclusion:** Significant association was found between the novel indices and CA success or failure. The novel index ER_NRMSE_ additionally shows good predictive power for single-procedure CA outcome.

## 1 Introduction

In contrast to the many studies that have analyzed the 12-lead electrocardiogram (ECG) for the study of atrial fibrillation (AF), relatively few have developed AF analysis tools for body-surface potential map (BSPM) signals. Despite the development of these 12-lead ECG based indices, to our knowledge, the use of ECG for AF in clinical practice is still limited to its diagnosis (Lankveld et al., 2014). The traditional 12-lead ECG was designed to capture mainly ventricular activity (VA), therefore, BSPM signals could harbor additional information from the atrial activity (AA) relevant for AF analysis.

Various studies have performed analyses of BSPM data for the study of AF. For example (Bonizzi et al., 2010), applied principal component analysis (PCA) to BSPM data and proposed two novel parameters derived from the resulting PCA mixing matrices to quantify complexity and stationarity in BSPM recordings, finding a significant inverse correlation between the two. It was additionally found that these parameters formed clusters for organized vs. disorganized AF, but no further clinical application was proposed by the authors. The study in (Di Marco et al., 2012) proposed four parameters to quantify spatial organization, variability, spectral concentration, and spectral variability of BSPM signals. They found that greater spatial organization was associated with reduced variability of spatial organization over time, and that lower spectral variability was associated with increased spectral concentration. However, the clinical impact of the parameters was not assessed. Later (Meo et al., 2018), proposed the non-dipolar component index (NDI), which was calculated as the residual variance not accounted for by the first three principal components (PCs) of concatenated TQ segments of BSPM signals. It was found that the NDI correlated with AF complexity and AF termination at the end of catheter ablation (CA) procedures. However, correlation with clinical CA outcome was not reported, and the NDI leaves unexploited the temporal variability of BSPM signals found to be indicative of AF organization in (Bonizzi et al., 2010; Di Marco et al., 2012). These parameters do however show promise for capturing information from BSPM signals relevant for the computational analysis of AF signals. Additionally, they are all linked in that they use PCA in the computation of their indices. However, insufficient attention has been paid to the temporal aspect of the data, with most of the parameters using concatenated TQ segments. While this method has merit as it eliminates the possibility of interfering VA, it precludes a temporal analysis, and we aim to address this with our novel indices.

Despite the above research, the use of BSPM for persAF treatment remains limited in clinical practice, and there are not many tools available for its efficient analysis. Apart from its use in electrocardiographic imaging (ECGI), the clinical advantage of BSPM over 12-lead ECG signal analysis for AF remains unclear. In this study, we draw on the above research to propose two novel indices also employing PCA: error ratio, normalized root-mean square error (ER_NRMSE_) and error ratio, mean absolute error (ER_ABSE_), which exploit spatiotemporal information in BSPM recordings. The indices make use of the full set of BSPM electrodes by measuring how well only a subset of electrodes can represent AA on all BSPM electrodes compared to the optimal PCA-representation. The indices also encapsulate the temporal variability of the AA using long-duration BSPM recordings. We hypothesized that when subsets can represent AA nearly as well as the optimal PCA-representation, the AF is more organized and stable with time, and thus more likely to respond well to CA treatment. When the optimal PCA representation is much better than the electrode subset representation, we hypothesized that this could be indicative of a disorganized AF that is more variable over time, manifested as greater spatial variability in the recorded AA on the BSPM vest. In this way, we aim to provide additional tools which efficiently utilize the large amount of data incurred when working with BSPM recordings and show their clinical relevance for persAF disease quantification through correlation with and prediction of single-procedure CA outcome. We first discuss the study population and BSPM signal acquisition and pre-processing. An overview of the novel indices and BSPM vest electrode subset selection required for their calculation is then given. The statistical cross-validation protocol used to evaluate their clinical relevance is then presented. Finally, the results, implications, and limitations of the study are discussed.

## 2 Methods

### 2.1 Study population

We studied a total of 13 patients admitted for CA of drug-refractory persAF. Their baseline characteristics are reported in Table 1. The CA endpoint was complete pulmonary vein isolation (PVI). Electrical cardioversion was performed on patients still in AF after PVI completion to restore sinus rhythm (SR). Patients were then monitored throughout a follow up period and divided into two groups: 1) SR and arrhythmia recurrence (AR), according to whether they experienced an AR within 6 months, but at least 2 months after undergoing CA. The study protocol was approved by the Lausanne University Hospital Human Research Ethics Committee, and all patients provided written informed consent.

**TABLE 1:** Study population baseline characteristics.

	*n* = 13
Sex (M/F)	8/5
Age, mean ± std, years	63 ± 8
Hypertension, *n*	6
Coronary artery disease, *n*	2
Heart Failure, *n*	3
Valvular Disease, *n*	3
Diabetes, *n*	2
Left ventricular ejection fraction, mean ± std, %	51 ± 13
Sustained AF duration, mean ± std, months	11 ± 13

std, standard deviation.

### 2.2 BSPM signal acquisition and preprocessing

**FIGURE 1:**
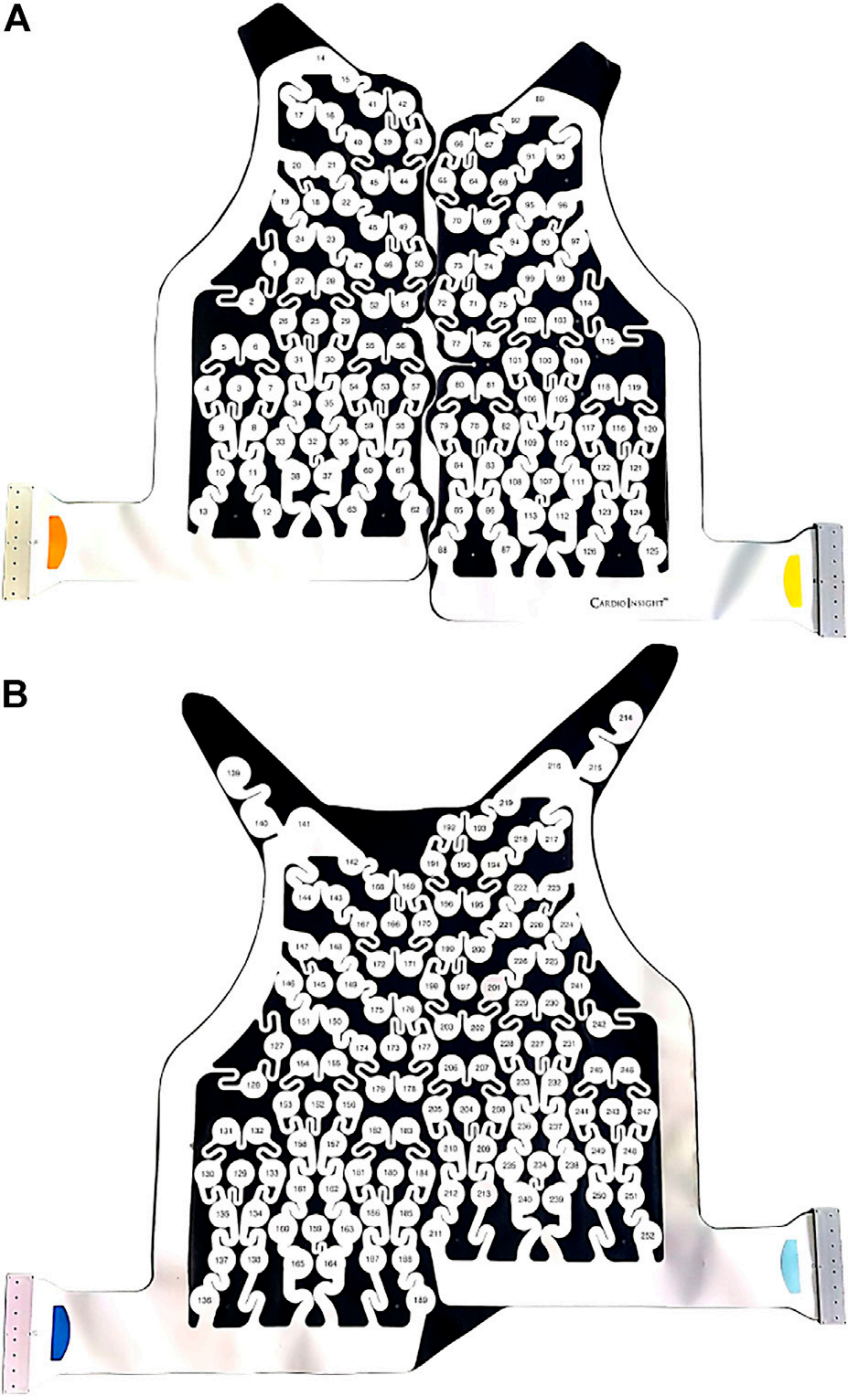
CardioInsight™ BSPM vest with 252 unipolar electrodes. The reference electrode is not shown. **(A)** Anterior part of vest with 126 electrodes. **(B)** Posterior part of vest with 126 electrodes.

**FIGURE 2:**
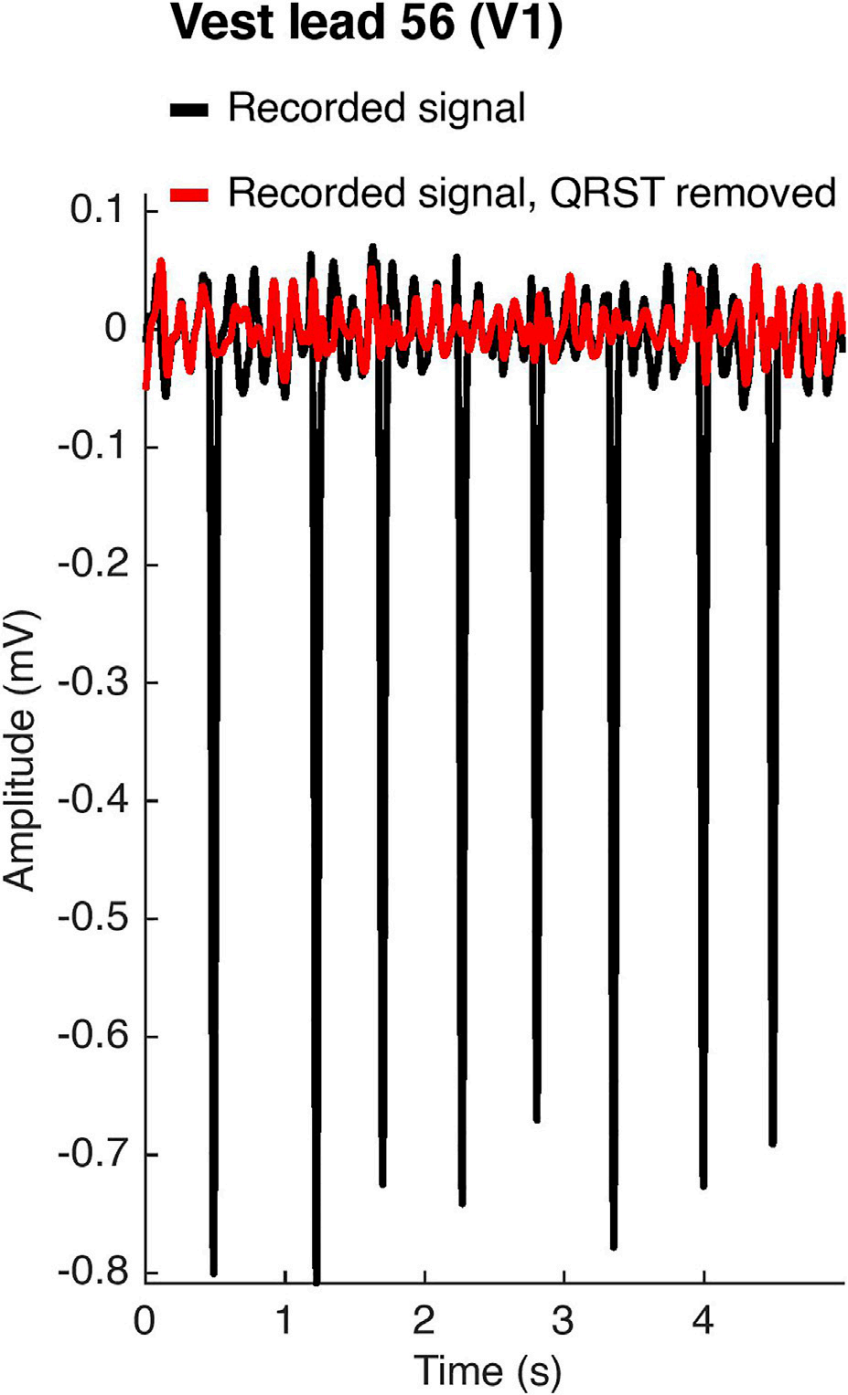
Atrial activity extraction. In black, the original recorded signal on vest lead 56, which corresponds to lead *V*_1_ of the standard 12-lead ECG configuration. In red, the signal after spatiotemporal QRST cancellation.

BSPMs were recorded with a 252-lead vest (CardioInsight™, Medtronic, MN, United States) at a sampling frequency of 1 kHz in persAF patients the day before undergoing CA at Lausanne University Hospital in Switzerland. A schematic of the vest is shown in Figure 1. Mean signal recording duration was 17 ± 4 min. Electrode contact and signal quality varied considerably over the course of the recording duration. Recordings were therefore visually inspected, and 1-min segments with good signal quality were extracted from the long duration recordings for further analysis. Remaining leads with poor signal quality were removed (up to 30 leads), and signals from high quality leads were used to estimate BSPM signals at the removed locations using interpolation. Recordings were then processed for removing baseline drift and high frequency noise (bandpass filter 1–30 Hz). R-peaks were detected and QRST delineation was performed in each lead using an open source ECG delineation toolbox (Pilia et al., 2021). In order to evaluate BSPM signals free of ventricular interference and enable a temporal analysis, the spatiotemporal method for QRST cancellation was applied (Stridh and Sornmo, 2001). This method, which operates on a single-beat, multi-lead basis, was chosen due to its exploitation of multiple leads and its tested performance (Langley et al., 2006). Clustering was applied to QRST complexes across all leads, so that the complexes used in each ensemble average had similar shapes. The extracted 1-min atrial activity BSPM segments (AA-BSPM) devoid of VA were then further normalized to have zero mean and unit variance, and low-pass filtered by a 10th order Butterworth filter with a cutoff frequency of 30 Hz to eliminate signal discontinuities introduced by spatiotemporal VA cancellation. An example of a BSPM signal before and after AA extraction is shown in Figure 2.

The power spectral densities (PSD) of all AA-BSPM segments were computed using a Welch periodogram (2-s Hamming window with a 4,096 Fast Fourier transform per window and 50% window overlap) to determine the body surface distribution of the atrial DF. The DF was defined as the highest peak in the power spectrum.

### 2.3 AA-BSPM reconstruction using a subset of BSPM electrodes

The purpose of this section is to describe how only a subset of vest electrode signals may be used to reconstruct the signals on all vest electrodes, as this is an important concept in the development of the novel indices ER_NRMSE_ and ER_ABSE_ (elaborated in Section 2.5). Each AA-BSPM segment may be represented by a matrix $X\in R^{m\times n}$ , with *m* consecutive samples in the rows, and *n* synchronously recorded signals in the columns, one from each BSPM vest electrode. Given an arbitrary subset of signals recorded on *k* vest electrodes, $S\in R^{m\times k}$ , *k* < *n*, the minimum least-squares transformation of the subset matrix *S* to approximate the full BSPM signal matrix *X* is given by:

$$\underset{A}{\mathrm{argmin}}\|X-SA{\|}_{F}^{2}$$ (1)

whose known solution is *A* = *S* ^+^ *X*, where (⋅)^+^ and ‖ ⋅‖_*F*_ denote the Moore-Penrose pseudoinverse and the Frobenius norm of a matrix, respectively. Then, to represent the full BSPM signal matrix *X* as accurately as possible, one should aim to find a subset *S* of vest electrode signals such that the AA-BSPM segment *X* is best represented by *S*, most commonly in terms of the Frobenius norm:

$$\underset{S}{\mathrm{argmin}}\|X-SS^{+}X{\|}_{F}^{2}$$ (2)

Finding the subset *S* is thought to be an NP-hard problem, with nk solutions, (Civril, 2014; Altschuler et al., 2016). Therefore, finding the optimal solution would involve searching the nk solutions, which for *n* and *k* of reasonable size is not feasible. The goal for electrode subset selection is then to find good, but not necessarily optimal subsets, and this will be discussed in the next section. An upper limit on the performance of a subset of *k* electrode signals for reconstructing the signals on all *n* electrodes is given by reconstruction using the first *k* principal components (PCs), since the explicit goal of PCA is to minimize the Frobenius reconstruction criterion. The PCs may be obtained efficiently by singular value decomposition (SVD) of *X*, *X* = *U*Σ*V* ^*T*^. The first *k* PCs are the columns of the matrix *U*_*k*_Σ_*k*_, where *U*_*k*_ contains the first *k* columns of *U*, and Σ_*k*_ contains the *k* × *k* upper-left portion of *Σ*. The corresponding rank-*k* reconstruction of *X* is given by:

$$X_{k}=U_{k}\Sigma_{k}V_{k}^{T}$$ (3)

where *V*_*k*_ contains the first *k* columns of *V*. Then the minimum approximation error $\|X-Q{\|}_{F}^{2}$ which can be attained by an arbitrary rank-*k* matrix is achieved when *Q* = *X*_*k*_. Note that each PC is a linear combination of the signals on all *n* electrodes of *X*. Therefore, the rank-*k* PCA solution *X*_*k*_ is distinct from reconstruction using a subset *S* of *k* vest electrode signals and serves only as an upper bound to compare how well a given *S* captures the information on all BSPM electrodes.

### 2.4 Electrode subset selection and comparison

The purpose of this section is to describe the different subsets of vest electrodes we used for the calculation of the novel indices ER_NRMSE_ and ER_ABSE_, which will be elaborated in the next section. We also describe the characterization of the vest electrode subsets in terms of their capacity to accurately represent AA-BSPM signal information on all vest electrodes and compare this to the optimal PCA representation, as this is an important concept for the understanding of the novel indices.

1) Sequential: This algorithm was first proposed by (Lux et al., 1978) and later used by (Guillem et al., 2008, 2009) for selecting electrode subsets from a wider array of body vest electrodes. As its name suggests, this sequential approach greedily chooses electrodes one after the other, at each step picking the column containing the electrode signal which minimizes the reconstruction error, i.e., Eq. 2. This algorithm is in no way guaranteed to be optimal, since at each step it only considers that one additional electrode must be selected, rather than considering the entire subset. However, it can be relatively efficient (Farahat et al., 2011; Altschuler et al., 2016) and in general has had good performance for electrode selection. Therefore, it was chosen for use in this study. We call SEQ_k_ the subset of *k* vest electrodes (columns) chosen sequentially from the BSPM signal matrix *X*, for *k* = 8 : 30 electrodes. The lower limit of *k* = 8 electrodes was chosen to equal the number of independent leads used in the standard ECG, and the upper limit of *k* = 30 electrodes was chosen as a result of suggestions that roughly 30 electrodes are necessary to accurately represent AA in AF (Guillem et al., 2009).

2) Standard ECG: A subset of BSPM vest electrodes closest to the positions of the six precordial leads plus two limb leads used in the standard 12-lead ECG was extracted. We refer to this subset, which contains eight electrodes, as ECG_8_.

3) Augmented ECG: Additionally, there have been suggestions that posterior electrodes may be desirable to better capture left atrial activity in AF. Therefore, a subset of vest electrodes closest to the augmented ECG suggested in (Petrutiu et al., 2009) consisting of the eight electrodes in the ECG_8_ subset plus three posterior electrodes, *V*_8_, *V*_9_, and *V*_10_ in the same horizontal plane as *V*_6_, was tested. We refer to this subset, which contains 11 electrodes, as ECG_11_.

Subsets were compared regarding how well they could represent the full BSPM signal matrix as follows. SEQ_8:30_, ECG_8_, ECG_11_, and PCA_8:30_ subset reconstructions were calculated for 5-s windows of each AA-BSPM segment. Given $\hat{X}$ the reconstruction (Eq. 2) and *X* the full BSPM signal matrix for a window, two error measures were calculated and averaged over all windows in a segment. The first was the normalized root-mean square reconstruction error (NRMSE), given by:

$$\text{NRMSE}=\frac{∥X-\hat{X}{∥}_{F}}{∥X{∥}_{F}}$$ (4)

The second was the mean absolute difference (ABSE) across all electrodes between the atrial DF on each vest electrode signal of *X* and $\hat{X}$ , where the DF was obtained as described at the end of the previous section:

$$\text{ABSE}=\frac{1}{L}\overset{l=L}{\underset{l=1}{\sum }}|DF\left(X\left(:,l\right)\right)-DF\left(\hat{X}\left(:,l\right)\right)|$$ (5)

where *L* = 252 vest electrodes in our case. The above measures were calculated on 5-s windows of the 1-min AA-BSPM segments extracted from each patient and then averaged over all windows, for $\hat{X}$ found for the ECG_8_, ECG_11_, and SEQ_8:30_ subsets determined for each window. Finally, for comparison, the above measures were calculated for $\hat{X}=X_{k}$ , the rank-*k* PCA reconstruction as described in Eq. 3 for *k* = 8 : 30.

### 2.5 Novel spatiotemporal indices ER_NRMSE_ and ER_ABSE_

The purpose of this section is to combine the concepts discussed in Sections 2.3, 2.4 to introduce the novel indices ER_NRMSE_ and ER_ABSE_. These indices quantify the capacity of a subset of BSPM vest electrodes to accurately represent the AA, and the atrial DF, respectively, on all BSPM electrodes over time, compared to the optimal PCA representation of the same rank as the electrode subset.

For the calculation of the indices, the 1-min AA-BSPM segments were divided into windows of 5-s as in Section 2.4. The SEQ_8_ and SEQ_11_ subsets were obtained for the first window, and reconstructions were obtained on subsequent windows using these subsets and the corresponding matrices *A* from Eq. 1 determined for the first window. Concretely, for the first 5-s window, the solution to Eq. 1 is $A^{1}=S_{k}^{1}X^{1}$ , where *X* ^1^ is the full BSPM signal matrix for the first window, and $S_{k}^{1}$ contains the signals on a subset of *k* vest electrodes. The reconstruction for the *i^th^* window *X* ^*i*^ is given by ${\hat{X}}^{i}=S_{k}^{i}A^{1}$ , where $S_{k}^{i}$ contains the signals of *X* ^*i*^ on the SEQ_8_/SEQ_11_ subset electrodes determined for the first 5-s window *X* ^1^, or the ECG_8_/ECG_11_ subset electrodes. In addition, the optimal rank-*k* (*k* = 8, 11) PCA reconstruction for each window, $X_{k}^{i}$ , was determined as previously described, using the first *k* PCs, which we refer to as PCA_k_. Then, the first index, ER_NRMSE_, is given by the ratio of the reconstruction error obtained using SEQ_8_, SEQ_11_, ECG_8_, or ECG_11_, and the optimal same-rank reconstruction obtained with PCA_8_ or PCA_11_:

$$ER_{NRMSE_{i}}=\frac{∥X^{i}-{\hat{X}}^{i}{∥}_{F}}{∥X^{i}-X_{k}^{i}{∥}_{F}}$$ (6)

The second index, ER_ABSE_, is given by the ratio of the mean-absolute error between the atrial DFs extracted on each electrode of the subset vs. PCA reconstructions:

$$ER_{ABSE_{i}}=\frac{\sum_{l=1}^{l=L}|DF\left(X^{i}\left(:,l\right)\right)-DF\left({\hat{X}}^{i}\left(:,l\right)\right)}{\sum_{l=1}^{l=L}|DF\left(X^{i}\left(:,l\right)\right)-DF\left(X_{k}^{i}\left(:,l\right)\right.)}$$ (7)

The above indices were calculated on each 5-s window following the first window of the 1-min AA-BSPM segments, then averaged across all windows, for one value per 1-min AA-BSPM segment. We chose to calculate these indices for the SEQ_8_ and SEQ_11_ subsets to allow a direct comparison with the standard and augmented ECG subsets, ECG_8_ and ECG_11_.

We hypothesized more organized, easier to treat forms of AF should have lower error ratios (ER), indicating stability in the AF dynamics between windows. This is because for lower ERs, the subset of electrodes chosen for the first window and *A* ^1^ permit a reconstruction of the *i^th^* window that is closer to the optimal PCA reconstruction of the same rank. As a comparison to another BSPM index utilizing PCA for AF analysis, we calculated the NDI proposed by (Meo et al., 2018), which was found to be useful for quantifying AF complexity, choosing patients eligible for AF ablation and assessing therapy impact. The NDI was calculated as the residual variance not accounted for by the first three PCs of each AA-BSPM window. Note also that this index uses only PCA and therefore is not dependent on any particular subset of electrodes.

### 2.6 Performance metrics and statistical analysis

The purpose of this section is to describe how the different electrode subsets introduced in Section 2.4 were compared in terms of their capacity to accurately represent AA-BSPM signal information on all vest electrodes compared to the optimal PCA representation. We also describe the methods used to quantify the relationship between the indices ER_NRMSE_, ER_ABSE_ and NDI, and single-procedure CA success rate.

Calculated values of continuous parameters are expressed as mean ± standard deviation. The statistical distributions of all parameters were checked using a Lilliefors test. Statistical inter-group differences were calculated as mean *p*-values across 3-folds, with 20% of the parameter values left out of each fold. One-way analysis of variance (ANOVA) was used for normally distributed data, or Wilcoxon’s rank sum test was used for non-normally distributed data. Statistical tests were performed across folds to reduce the likelihood of chance group differences due to a small data set, and statistical significance was considered for *p*-values less than 0.05. For comparing the different electrode subsets in terms of their capacity to accurately represent AA-BSPM segments, we checked for statistical differences of the NRMSE and ABSE parameters calculated using the different electrode subsets (SEQ_k_, ECG_8_, ECG_11_), or using the corresponding number of PCs (PCA_k_). For comparing the relationships between single-procedure CA outcome and ER_NRMSE_, ER_ABSE_, and NDI, we checked for statistical differences between these indices calculated for AA-BSPM segments associated with AR and SR outcome groups.

For indices ER_NRMSE_, ER_ABSE_, and NDI displaying statistically significant differences between groups, univariate logistic regression classifiers were used to test their predictive power for single-procedure CA outcome. We used group-wise 3-fold cross-validation (CV) to ensure that indices calculated from different AA-BSPM segments of the same patient were only assigned to either the train or test set (80/20) for each fold. The resulting receiver operating curves (ROC) were analyzed to obtain area under the curve (AUC) to compare the predictive power of each index. We reported the AUC for AA-BSPM segment-wise classification as mean ± standard deviation over all CV folds, and the sensitivity and specificity were reported for the optimal classification threshold value determined through ROC analysis. The sensitivity and specificity were the fraction of true positive and true negative cases correctly identified, respectively, where AR was considered a positive case, and SR was considered a negative case.

## 3 Results

### 3.1 Study population

At the time of this study, clinical outcome information was available for 11 patients, six of whom experienced an AR (55%) (3.4 ± 0.9 months post-CA). Therefore, only BSPM data from 11 patients were available for the part of the study associating the novel indices to CA outcome. Patients experiencing an AR were offered repeat procedures, however, in this study, only signals recorded prior to the first procedure and associated clinical outcomes were analyzed. Seven high quality 1-min AA-BSPM segments were extracted from the long duration BSPM recordings of each patient. Therefore, 91 1-min AA-BSPM segments were available for the analysis with results described in Section 3.2, and 77 AA-BSPM segments for the results described in Sections 3.3, 3.4, with each segment associated with an SR or AR outcome.

### 3.2 AA-BSPM reconstruction with BSPM electrode subsets

**FIGURE 3:**
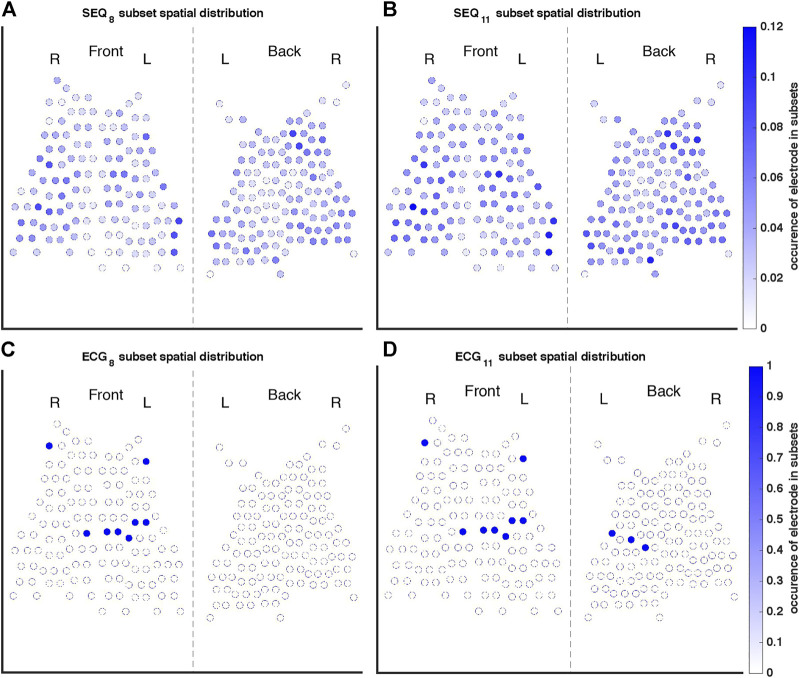
Occurrence of each electrode in the **(A)** SEQ_8_, **(B)** SEQ_11_, **(C)** ECG_8_, and **(D)** ECG_11_ subsets. The color of each electrode indicates its occurrence in all of the subsets used to calculate reconstruction performance measures, where, for example, one indicates the electrode was present in every subset tested, and 0.1 indicates the electrode was present in 10% of subsets.

A representation of the spatial distributions of the SEQ_8_, SEQ_11_, ECG_8_, and ECG_11_ subsets used for AA-BSPM reconstruction is shown in Figure 3. For each subset type, the color of each electrode represents its occurrence in all the subsets used to calculate NRMSE and ABSE, that is, in what ratio of the tested subsets the electrode was included. It can be seen in Figures 3A,B, that for SEQ subsets, both anterior and posterior electrodes were included in the subsets, with certain torso regions (upper and lower) being less represented in the subsets, while the mid-section regions were in general more represented. For the ECG subsets, the same subset of electrodes was applied to each 5-s window of AA-BSPM data, as seen in Figures 3C,D.

**FIGURE 4:**
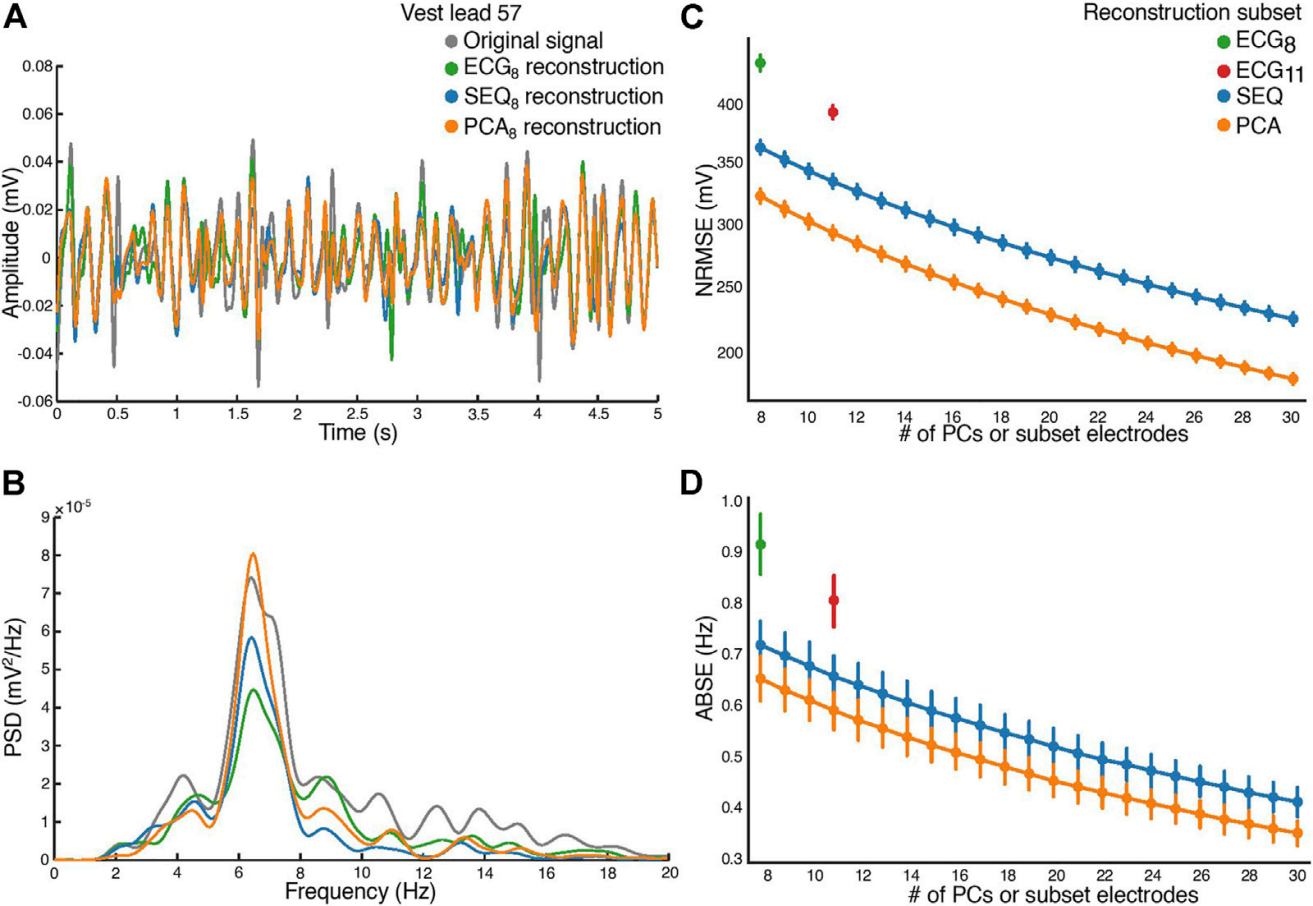
Atrial activity reconstruction. **(A)** A 5-s window of the original recorded (in grey) and reconstructed signals, for vest electrode 57, close to *V*_1_. Reconstruction with the ECG_8_ subset (green), SEQ_8_ subset (blue), and PCA_8_ (orange). **(B)** The corresponding power spectral densities (PSD) of the original recorded and reconstructed signals. **(C)** Normalized root-mean square reconstruction error (NRMSE), and **(D)** mean absolute error (ABSE), as a function of number of principal components or vest electrodes used in subset for reconstruction.

The similarity between reconstructed and original BSPM signals is shown in Figure 4. A 5-s window of a signal recorded on vest lead 57, close to the position of precordial lead *V*_1_, as well as its optimal least-squares reconstructions using the ECG_8_ and SEQ_8_ subsets associated with the window and its PCA_8_ reconstruction are demonstrated in Figure 4A. It can be seen that morphological characteristics of the signal were mostly captured in the reconstructed signals, however, the amplitude of the recorded signal was not perfectly reconstructed. The power spectral densities of the original and reconstructed signals are shown in Figure 4B. It can be seen that the DF was correctly captured on this electrode. The NRMSE and ABSE as a function of number of electrodes included in the subsets are shown in Figures 4C,D, taken as a mean across all patients, with error bars representing the 95% confidence interval. In addition, the PCA_k_ reconstruction obtained for *k* PCs equaling the number of electrodes is shown. As expected, reconstructions using ECG_8_ and ECG_11_ subsets had higher NRMSE and ABSE values than reconstructions using SEQ_8_ and SEQ_11_, and this difference was statistically significant (*p* < 0.01). In addition, it can be seen that PCA_k_ reconstruction consistently performed better than SEQ_k_ reconstruction, and this difference was also statistically significant for *k* = 8 : 30, (*p* < 0.01). An example showing the DF of each electrode for original recorded AA-BSPM and AA-BSPM using ECG_8_, SEQ_8_, and PCA_8_ reconstruction is shown in the Supplementary Materials, where it can be seen that spatial differences in the original DFs at different parts of torso were mostly captured by SEQ_8_ and PCA_8_ reconstruction, and to a lesser extent by ECG_8_ reconstruction.

### 3.3 BSPM AF spatiotemporal indices and CA outcome

**FIGURE 5:**
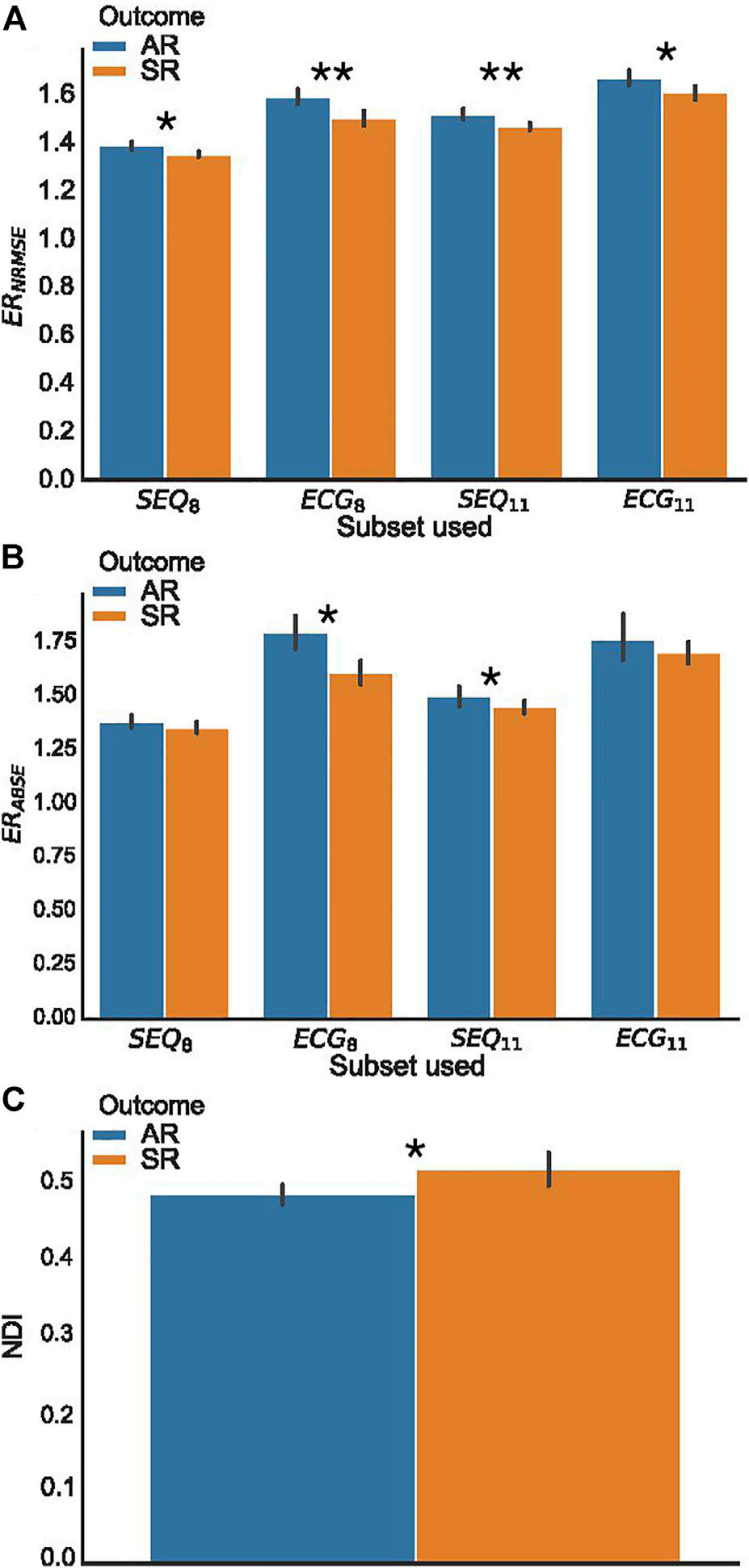
BSPM analysis indices, grouped according to clinical outcome. **(A)** Error ratio NRMSE to PCA (ER_NRMSE_); **(B)** Error ratio ABSE to PCA (ER_ABSE_); **(C)** Non-dipolar component index (NDI). Significant differences between AR and SR outcome means are indicated by asterisks. (*p < 0.05, **p < 0.01.).

For the analysis of ER_NRMSE_ and ER_ABSE_, only the indices calculated using SEQ_8_, SEQ_11_, PCA_8_, and PCA_11_ were used, to allow for comparison with ECG_8_ and ECG_11_. Shown in Figure 5 are the (A) ER_NRMSE_, (B) ER_ABSE_, and (C) NDI values according to CA outcome. The associated mean ± standard deviation values of the indices are shown in Table 2. There was a statistically significant difference in the NDI value between the SR and AR groups, with NDI greater in the SR group. Greater ER_NRMSE_ values were associated with AR following single-procedure CA, while lower ER_NRMSE_ values were associated with SR, for all electrode subsets tested. Finally, for ER_ABSE_, greater values were again observed for AR than SR, with statistical significance only for the ECG_8_ and SEQ_11_ subsets.

**TABLE 2:** ER_NRMSE_, ER_ABSE_, and NDI values by vest electrode subset (not applicable for NDI) and single procedure CA outcome, expressed as mean ± standard deviation. *p*-values were computed as means across three folds of data, in which a *p*-value for the statistical significance between index values by outcome was computed using 80% of the AA-BSPM segments in each fold.

Subset	Outcome	ER_NRMSE_	*p*-value	ER_ABSE_	*p*-value	NDI	*p*-value
SEQ_8_	SR	1.349 ± 0.034	0.010	1.352 ± 0.078	0.294	0.517 ± 0.070	0.018
	AR	1.385 ± 0.053		1.38 ± 0.096		0.484 ± 0.041	
ECG_8_	SR	1.499 ± 0.091	0.008	1.607 ± 0.171	0.045		
	AR	1.589 ± 0.102		1.796 ± 0.253			
SEQ_11_	SR	1.464 ± 0.041	0.002	1.448 ± 0.093	0.033		
	AR	1.515 ± 0.075		1.497 ± 0.159			
ECG_11_	SR	1.605 ± 0.082	0.037	1.700 ± 0.150	0.107		
	AR	1.667 ± 0.100		1.760 ± 0.351			

### 3.4 Predictive power of spatiotemporal indices for single-procedure CA outcome classification

**FIGURE 6:**
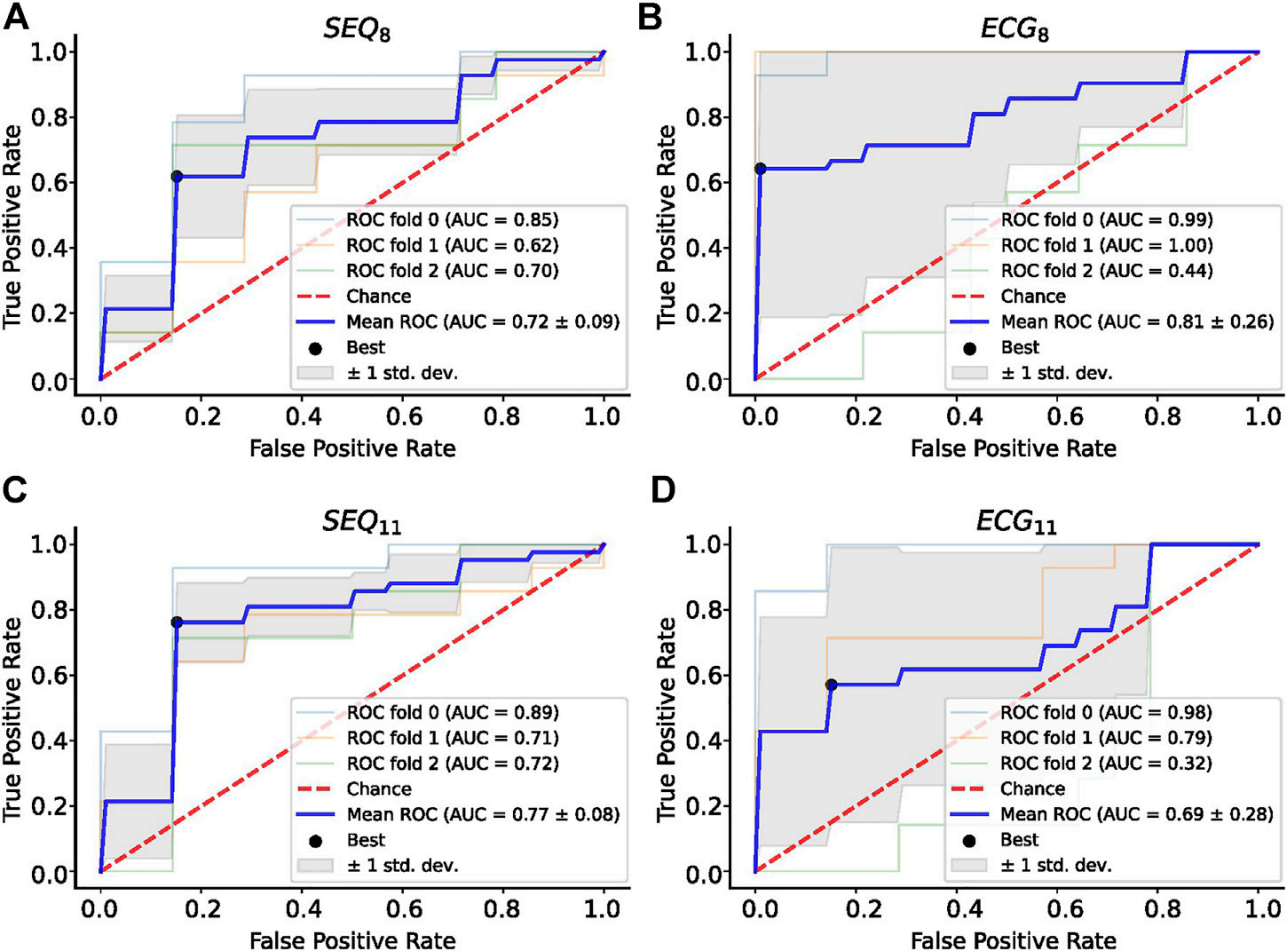
ROC Analysis for ER_NRMSE_ for predicting CA outcome calculated with **(A)** SEQ_8_; **(B)** ECG_8_; **(C)** SEQ_11_; and **(D)** ECG_11_ vest electrode subsets. The optimal tradeoff between true positive rate and false positive rate is indicated by a black dot.

A summary of the ROC analysis of the NDI, ER_NRMSE_, and ER_ABSE_ parameters for prediction of CA outcome is shown in Table 3. Note that sensitivity and specificity values are shown for segment-wise classification. It can be seen that ER_NRMSE_ displayed the most consistent performance across folds and electrode subsets, with AUC = 0.77 ± 0.08, sensitivity = 76.2%, and specificity = 84.8% for ER_NRMSE_ calculated with the SEQ_11_ electrode subset. Despite the associations between NDI and ER_ABSE_ and CA outcome, the predictive performances of these indices were not as consistent as for ER_NRMSE_. The ROC curves associated with ER_NRMSE_ calculated for each subset are shown in Figure 6. The ROC curves associated with NDI and ER_ABSE_ (for statistically significant subsets) are shown in the Supplementary Materials.

**TABLE 3:** Predictive power of BSPM indices for CA outcome, for each of the tested subsets. Sensitivity and specificity indicate the rate of detection of arrhythmia recurrence and sinus rhythm 6 months post single-procedure CA, respectively. AUC, area under the curve.

	AUC (mean ± std)		Sensitivity (%)		Specificity (%)	
NDI	0.37 ± 0.45		33		98.9	
	ER_NRMSE_	ER_ABSE_	ER_NRMSE_	ER_ABSE_	ER_NRMSE_	ER_ABSE_
SEQ_8_	0.72 ± 0.09	-	61.9	-	84.8	-
ECG_8_	0.81 ± 0.26	0.76 ± 0.23	64.3	57.1	98.9	98.9
SEQ_11_	0.77 ± 0.08	0.52 ± 0.29	76.2	28.6	84.8	98.9
ECG_11_	0.69 ± 0.28	-	57.1	-	84.8	-

**FIGURE 7:**
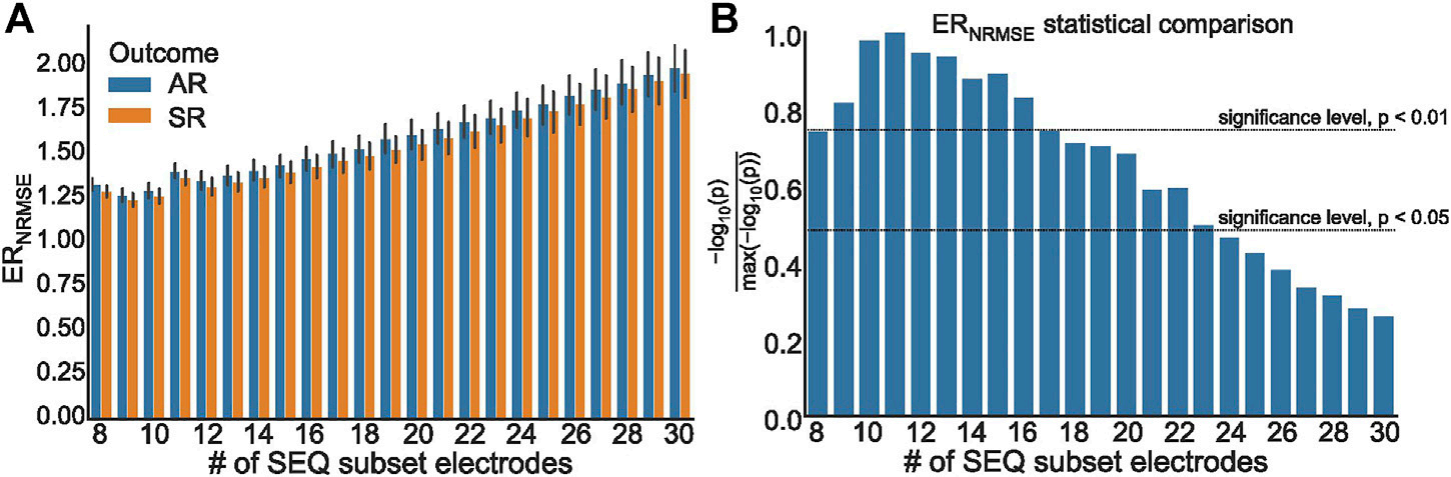
**(A)** ER_NRMSE_ values grouped according to clinical outcome, calculated with SEQ_8:30_ subsets; **(B)** Representation of *p* values associated with statistical comparison of ER_NRMSE_ mean values for AR and SR patient groups. The *p* = 0.05 and *p* = 0.01 significance levels are indicated by dashed lines.

**FIGURE 8:**
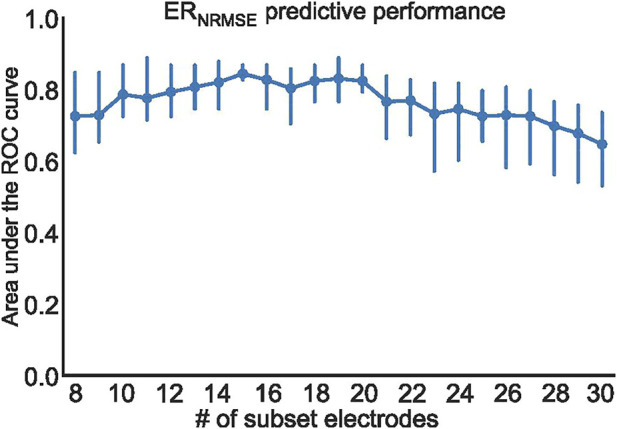
Mean and standard deviation of the area under the ROC curve (AUC) computed using three cross-validation folds, for ER_NRMSE_ performance for predicting CA outcome when calculated with SEQ_8:30_ electrodes.

To further test the efficacy of ER_NRMSE_, we repeated the statistical comparison and predictive performance analysis for SEQ subsets with 8–30 electrodes, to see whether performance changed for different numbers of electrodes included in the SEQ subset. The results are shown in Figures 7, 8. It can be seen in Figure 7A that the ER_NRMSE_ was greater in the AR group than SR group when calculated with all SEQ_8:30_ subsets. However, this difference was only statistically significant for ER_NRMSE_ calculated with SEQ_8:24_ subsets, as shown in Figure 7B, with the *p*-values from significance testing transformed as $\frac{-log10\left(p\right)}{\max\left(-log10\left(p\right)\right)}$ to allow for a graphical representation. In Figure 8 are shown AUC values associated with the ROC analysis of the ER_NRMSE_ calculated with SEQ_8:30_ subsets. It can be seen that the AUC increased, and variance of the AUC decreased, for up to 15 electrodes included in the SEQ subset. For more than 15 electrodes, the AUC generally decreased, and variance of the AUC increased.

## 4 Discussion

In this study, we developed two novel, fully spatiotemporal indices for the efficient processing of long-duration BSPM signals collected from patients with persAF. The use of spatiotemporal ventricular activity cancellation rather than short, nonconsecutive TQ segments allowed the incorporation of a temporal component in the analysis. By combining PCA and the temporal component, a true spatiotemporal characterization was achieved. To the best of our knowledge, this is the first study to propose indices exploiting temporal irregularity in long-duration BSPM recordings for persAF analysis, with a view to predicting AR following single procedure CA. The selection of which electrodes to use for the calculation of the novel indices can be automatically performed using the sequential subset selection method, or can be adapted for different subsets of electrodes, such as the standard or augmented ECG lead configurations. Finally, in our study, we have investigated the relationship between the proposed novel indices and their correlation with and predictive power for CA outcome. We found that a mean AUC of up to 0.8 may be achieved for predicting arrhythmia recurrence in persAF patients who underwent single-procedure CA for the novel index ER_NRMSE_.

### 4.1 Electrode subset capacity to represent AA-BSPM

PCA-based indices have been used extensively in ECG signal processing (Castells et al., 2007), with applications including extraction of atrial fibrillatory waves, quantification of AF spatial complexity and organization, and efficient analysis of BSPM data. In this study, we have included a framework for understanding how PCA-based reconstruction of AA-BSPM signals compares to reconstruction using a subset of vest electrode signals. The use of vest electrode reconstruction was based on previous studies, which have demonstrated that the full BSPM signal matrix may be projected onto a smaller matrix containing only a subset of BSPM vest electrode signals (Lux et al., 1978; Guillem et al., 2008, 2009; Feng et al., 2019). The resulting reconstruction error between the original and projected matrices has been shown to depend on the number of electrodes included in the subset, as well as the type of BSPM signals. For example, in (Guillem et al., 2009), it was found that with the same number of electrodes, reconstruction error was lower for ventricular than atrial activity. In our study, as the number of electrodes included in the ECG or SEQ subsets increased, the NRMSE and ABSE both decreased, in line with results from (Guillem et al., 2009). We also showed for the first time in our study the same trend for optimal PCA reconstruction. It can be noted that for all numbers of electrodes tested, there is a greater overlap between the ABSE values than NRMSE values between the different subset types. This could be because the reconstruction method used optimizes for NRMSE, and not ABSE.

Regarding the comparison of different BSPM electrode subsets, it was found that SEQ subsets more accurately represent BSPM signal data than standard and augmented ECG subsets in both signal domains, manifested through lower NRMSE and ABSE values for both *k* = 8 and *k* = 11 electrodes included in the vest subset. This reinforces the idea that standard ECG electrode configurations are not optimal in terms of accurately representing the full BSPM signal matrix. Since results from previous works have suggested that the addition of posterior electrodes may be useful to better reflect left atrial activity in AF (Ihara et al., 2007; Petrutiu et al., 2009; Buttu et al., 2013; Guillem et al., 2013), we did include the augmented ECG_11_ subset. Interestingly, it was found that the decrease in reconstruction error between the ECG_8_ and ECG_11_ subsets was greater than the decrease in error between the SEQ_8_ and SEQ_11_ subsets, lending support to the argument that the addition of carefully positioned posterior electrodes may indeed be beneficial for representing AA-BSPM data. Additionally, it was found that PCA-based reconstruction performs better than electrode subset based reconstruction, in both the temporal and frequency domains. This is to be expected, since PCA explicitly optimizes for the reconstruction criterion. However, this remains an important result since to our knowledge, the gap between electrode subset and PCA reconstruction had not been previously investigated. We hypothesized that this gap may contain useful information related to AF signal analysis.

### 4.2 Statistical comparison of novel indices

The finding in our study that ER_NRMSE_, ER_ABSE_, and NDI all show statistically significant differences between AA-BSPM segments associated with AR or SR outcomes demonstrates that each of these indices shows some potential to be used as computational tools for AF disease management. Since calculation of ER_NRMSE_ and ER_ABSE_ required selection of a subset of vest electrodes, these indices were calculated for standard and augmented ECG subsets (ECG_8_ and ECG_11_, respectively), as well as for sequentially chosen subsets (SEQ_8_ and SEQ_11_), which differ between AA-BSPM segments, to test the robustness of the indices with respect to both which and how many electrodes were included in the subsets.

Many previous studies have investigated capturing AF information using surface recorded ECG signals, however, most often using a single or limited number of leads. This prevents the exploitation of the spatial diversity of multi-lead ECGs and is dependent on the available electrode signal containing information representative of the underlying AF. Since electrode placement cannot be exact, and patient anatomy varies widely, this is not always guaranteed. Further, it has already been shown that inclusion of multiple leads is beneficial, leading to greater correlation between calculated 12-lead ECG indices and AF complexity and outcomes in (Meo et al., 2013; Zarzoso et al., 2016). The idea in this study was therefore to add to the limited number of BSPM indices for AF analysis, integrating both temporal and spatial information.

In (Bonizzi et al., 2010), it was shown that reconstruction error using the PCA rank-3 approximation of TQ segments of BSPM signals recorded during AF was capable of separating AA signals into clusters based on levels of AF organization, with greater reconstruction error corresponding to higher AF complexity and lower stationarity. Later, (Di Marco et al., 2012), using PCA-based indices, found that higher spatial organization, indicating easier to treat forms of AF, was correlated with more temporally stable atrial activation patterns. We hypothesized that lower ER_NRMSE_ values would therefore be observed among SR patients, indicating more stability in the AF dynamics between BSPM windows, and this was indeed the case. The finding that ER_NRMSE_ displayed greater values in AR AA-BSPM segments than SR segments for all four electrode subsets lends support to its use as a robust index for predicting single-procedure AF outcome. Further, also in (Di Marco et al., 2012), it was found that greater temporal variability was associated with lower spectral concentration. Therefore, we hypothesized again that lower ER_ABSE_ values would be observed for SR patients. While the ER_ABSE_ calculated for AR patients was greater than that for SR patients, this difference was only statistically significant for two of the electrode subsets tested, potentially making it less robust than ER_NRMSE_. This lower robustness could relate to the finding discussed above that there was a greater overlap in ABSE values calculated for subset vs. PCA reconstruction.

The NDI was unexpectedly found to be greater for SR segments than AR segments, meaning that the remaining variance unexplained by the first three PCs of the BSPM signal data was on average greater for SR segments than AR segments. This is contrary to the result in (Meo et al., 2018), which found smaller NDI values in concatenated TQ segments of BSPM data collected from patients with successful procedural CA outcome. Several key differences in our study could explain the contradictory results, including our use of longer duration AA-BSPM segments rather than concatenated TQ segments. Additionally, the study in (Meo et al., 2018) compared NDI values calculated for procedural outcomes, while in this study we used single-procedure clinical outcomes. Future studies testing the NDI may shed light on this discrepancy.

### 4.3 Assessment of clinical impact of novel indices

The clinical impact of the indices tested in this study depends not only on their association with CA outcomes but also their ability to predict CA outcomes. Therefore, we tested classification performance of univariate logistic regression classifiers for ER_NRMSE_, ER_ABSE_, and NDI. The use of group-wise CV was important to ensure AA-BSPM segments extracted from the same patient were included only in either the train or test set of each fold. Reporting both mean and standard deviation values of the AUC for each classifier also gave an indication of the CA outcome prediction model variance for different folds of the data. These were important features of our methodology considering the small-size of our data set.

Importantly, only ER_NRMSE_ displayed consistent results in predictive power across tested vest electrode subsets. The AUCs for this index calculated with each subset (SEQ_8_: 0.72 ± 0.09, ECG_8_: 0.81 ± 0.26, SEQ_11_: 0.77 ± 0.08, ECG_11_: 0.69 ± 0.28) were as good or better than the AUC associated with NDI (0.69), found in (Meo et al., 2018), and the model variance was not reported in their study. Additionally, the (Meo et al., 2018) study also tested prediction performance for NDI combined with clinical parameters, achieving an AUC of 0.7. Our results were also as good or better than those described in (Lankveld et al., 2016), in which predictive performance varied from AUC = 0.76 ± 0.15 for 12-lead ECG derived complexity parameters alone to AUC = 0.79 ± 0.13 for ECG plus clinical parameters. Additionally, the results were in line with those obtained in (Zeemering et al., 2018), for which an AUC of 0.66, 95% confidence interval [0.64–0.67] was obtained for the best ECG parameter studied (dominant atrial frequency in lead II). The study also reported better performance when several ECG parameters were combined (AUC 0.78 [0.76–0.79]), and best performance for combining ECG plus clinical parameters (AUC = 0.81 [0.79–0.82]). It is important to note that the variance across CV folds was less for ER_NRMSE_ calculated with SEQ subsets compared to ECG subsets, and in general lower than model variances reported in other studies. This result was also found to be true in our additional analysis calculating ER_NRMSE_ with SEQ_8:30_ electrodes, finding high predictive performance for nearly all numbers of electrodes included in the subset. That ER_NRMSE_ showed the best results and lowest variance when calculated with SEQ electrode subsets could indicate that when working with BSPM signals, an informed patient or segment specific selection of subset electrodes may be useful, for example using the sequential algorithm. This is logical given the nonstationary nature of BSPM recordings in AF. This was further supported by the lack of a clear pattern of specific electrodes included in the SEQ subsets as seen in Figure 3; rather, certain regions of the vest contain electrodes picked by the sequential algorithm more often than other regions. The finding that specificity was generally higher than sensitivity across all indices tested could indicate that this index would be more useful for selecting which patients would be most likely to benefit from CA, as opposed to selecting those least likely to benefit. Note that specificity was also higher than sensitivity for the NDI in the original study (Meo et al., 2018). The other two indices appear less robust to predict CA outcome, with low AUC and sensitivity values for NDI. For ER_ABSE_, the results appear more promising, but still displaying relatively low sensitivity and more variable AUC values across CV folds than ER_NRMSE_.

These results all point towards the potential value of ER_NRMSE_ as a clinically useful tool that could be used to analyze BSPM data in persAF. If confirmed by future studies, the use of ER_NRMSE_ could be important in assisting in prediction of successful CA outcomes, which would be useful for improving informed decision making regarding treatment for persAF. This would be particularly important given the low success rate of CA for treating persAF. Finally, the added clinical value of using BSPM data in AF remains unclear, as evidenced by its being largely limited to research use (Salinet et al., 2021). However, if indices calculated using BSPM data, such as ER_NRMSE_, could be shown to be consistently associated with and good predictors of CA outcomes, this could confirm the validity of using BSPM data for AF analysis.

### 4.4 Limitations

The study population, at 13 patients, and 11 patients with clinical outcome data, was small; however, the effort in obtaining BSPM recordings is considerable, due to the high number of electrodes which are used. The use of group-wise CV on segments of data extracted from each patient did however permit a robust analysis using this data set. In addition, the randomized inclusion criteria enhances our conclusions on BSPM AF characterization, though this may not be representative of the general characteristics of a wider population. Given the variation in experimental set up and parameters used, comparing with parameters from previous studies was challenging, and a more systematic study would be required for integration of the contributions of this work into clinical practice. Further, the small size of the population precluded the analysis of correlation with clinical indices, or whether the proposed indices could be combined with clinical indices for predicting CA outcome, as it has been shown previously in several studies that combining ECG-based indices with clinical parameters yields the best clinical performance (Lankveld et al., 2016; Meo et al., 2018; Zeemering et al., 2018). Additionally, the follow-up duration was relatively limited, and again due to the small study size, the impact of anti-arrhythmic medications could not be assessed since these were used by most patients. Finally, while we have shown the usefulness of the proposed indices for predicting CA outcome, we were unable to examine these indices for quantifying AF complexity due to a lack of available simultaneous intracardiac recordings.

### 4.5 Conclusion

In this study, we have proposed two novel indices for AF analysis with BSPM signals, ER_NRMSE_ and ER_ABSE_. We have shown clinical applicability by demonstrating correlation between the novel indices and single-procedure CA outcome and also promising outcome prediction performance. However, only ER_NRMSE_ values were found to be statistically greater for the AR patient group than SR patient group, and demonstrate consistently high CA outcome predictive performance when calculated with ECG subsets, and independent of the number of electrodes included in the SEQ subset used for its calculation. These results, combined with previous studies also employing PCA-based methods, suggest that continued study of BSPM signals for AF analysis is warranted.

## Data Availability

The original contributions presented in the study are included in the article/Supplementary Materials, further inquiries can be directed to the corresponding author.
