# Supplementary material for: Novel spatiotemporal processing tools for body-surface potential map signals for the prediction of catheter ablation outcome in persistent atrial fibrillation
Source: Front Physiol. 2022 Sep 29;13:1001060. doi: 10.3389/fphys.2022.1001060 (PMC9557152; doi:10.3389/fphys.2022.1001060)
Supplement: Supplementary file 1 [file Table1.DOCX]

Supplementary Materials:


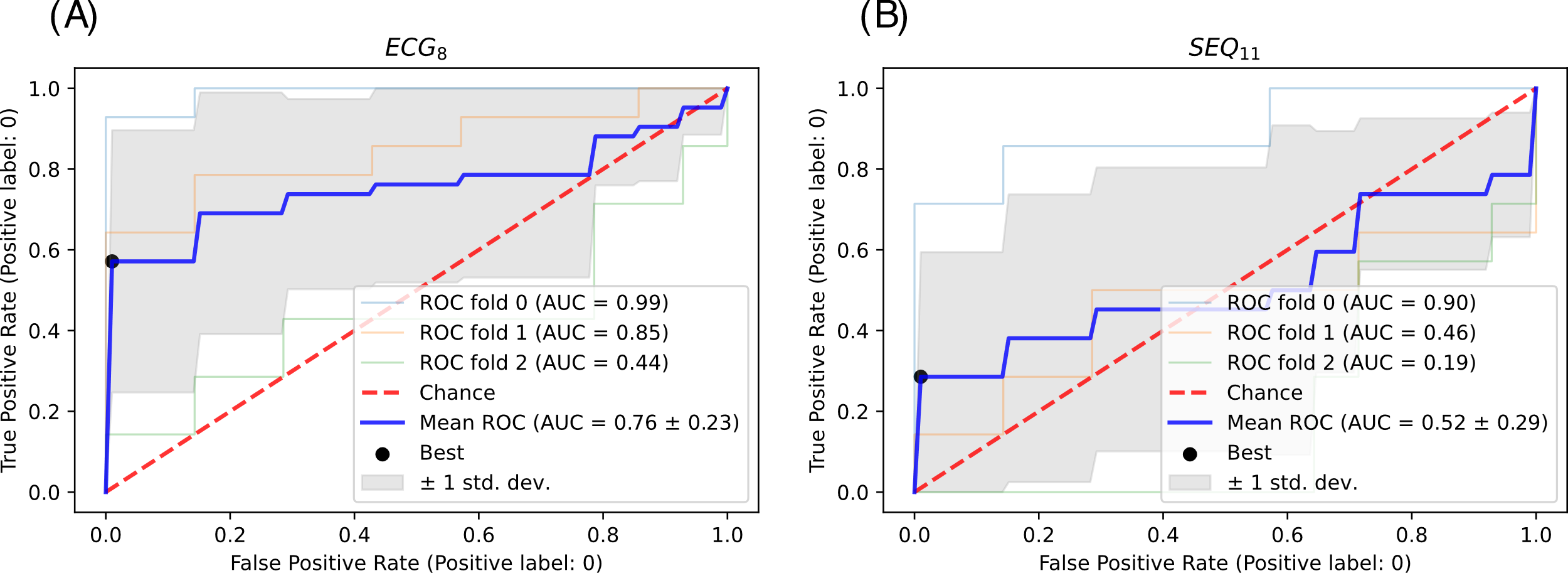


Figure 1: ROC analysis for ER_ABSE_ for predicting CA outcome, calculated with (A) ECG_8_ and (B) SEQ_11_ vest electrode subsets. The optimal tradeoff between true positive rate and true negative rate is indicated by a black dot. Note, no statistically significant difference between the outcome groups was found for the index calculated with SEQ_8_ and ECG_11_ subsets, so an associated ROC analysis was not performed.


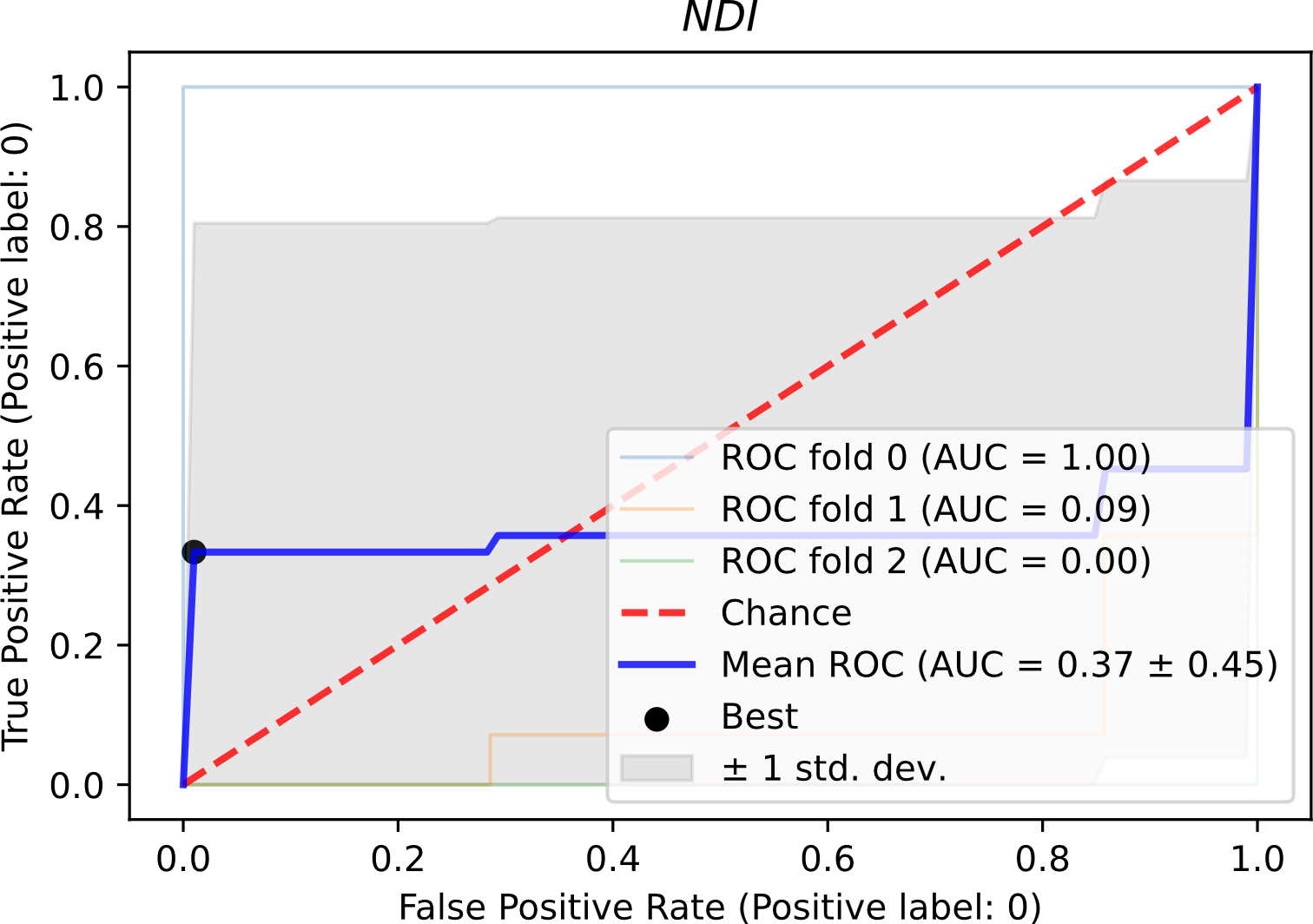


Figure 2: ROC analysis for NDI for predicting CA outcome. The optimal tradeoff between true positive rate and true negative rate is indicated by a black dot.


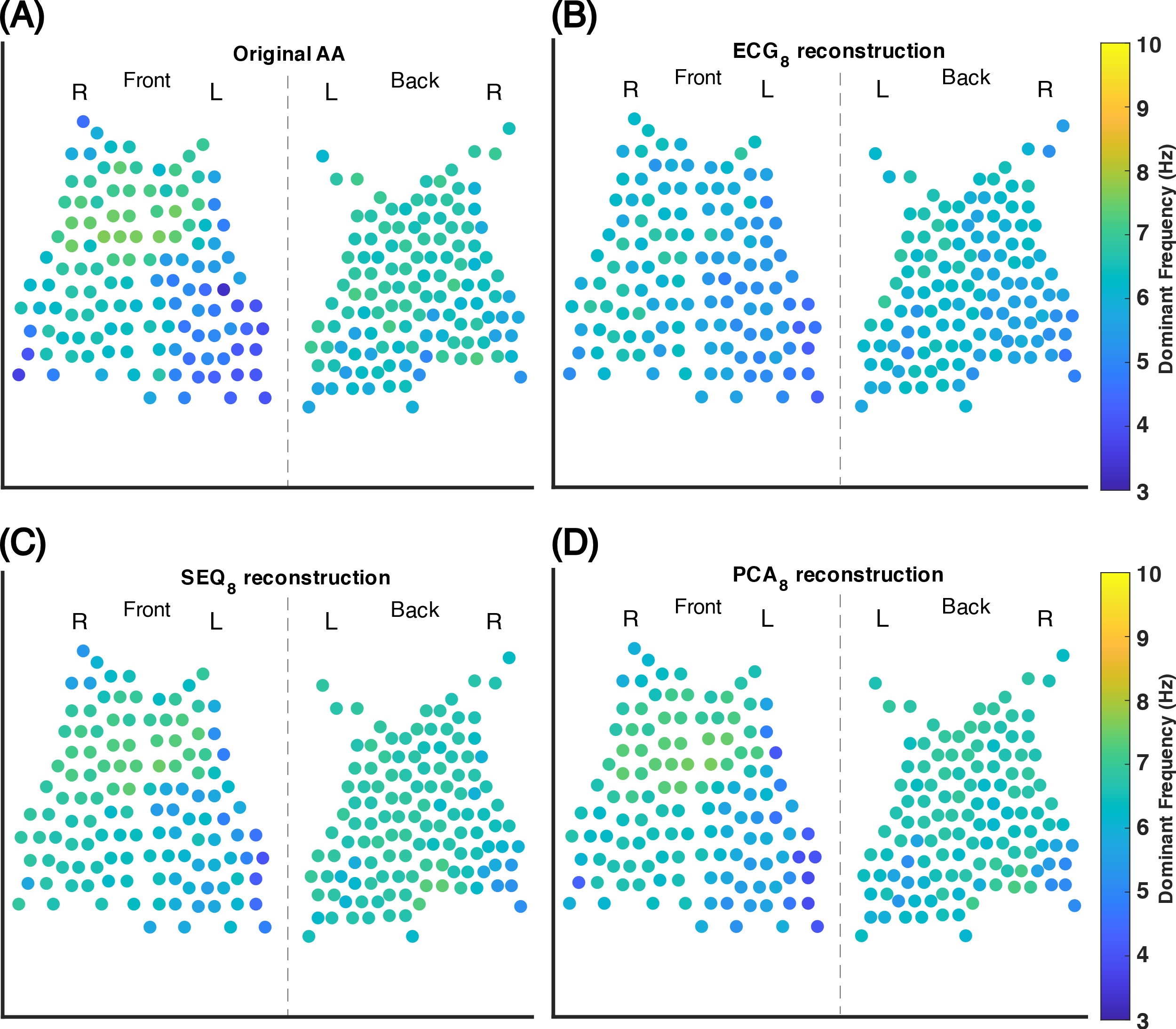


Figure 3: Example of vest atrial dominant frequency (DF) reconstruction for one patient. Each figure shows the dominant frequency of the AA-BSPM segment on each electrode. Each dot in the figures represents one vest electrode, either on the front (left half) or back (right half) of each figure. The right and left sides of each torso face are indicated by ‘R’ and ‘L’. Shown are (A) the original recorded AA-BSPM DFs, (B) AA-BSPM DFs with ECG_8_ reconstruction, (C) AA-BSPM DFs with SEQ_8_ reconstruction, and (D) AA-BSPM DFs with PCA_8_ reconstruction.
